# Supplementary material for: Surface Treatment Effects on the Mechanical Properties of Silica Carbon Black Reinforced Natural Rubber/Butadiene Rubber Composites
Source: Polymers (Basel). 2019 Oct 27;11(11):1763. doi: 10.3390/polym11111763 (PMC6918317; doi:10.3390/polym11111763)
Supplement: Supplementary file 1 [file polymers-11-01763-s001.pdf]

Supplementary Materials: The production of natural rubber (NR) is derived from the rubber tree, which is solidified by acidification, tableted, and smoked to make ribbed smoked sheet (RSS). Butadiene rubber (BR) (cis-1, 4-polybutadiene) is prepared from 1, 3-butadiene by solution polymerization in a nickel catalytic system. In this study, a nature rubber ribbed smoked sheet (RSS1) was produced in Vietnam, with an NR molecular weight of 1.3 x10^5 to 1.5x10^5 g/mol. BR was produced in Sinopec Yanshan Petrochemical Company, with BR molecular weight of around 9000 g/mol. The detailed characteristics of both NR and BR was provided as Table S1.

Rice husk ash (RHA) mainly consists of amorphous silica and residual carbon black. In addition to silica and carbon, there is also a small amount of nonvolatile inorganic constituents such as oxides of the alkali elements (potassium, magnesium, sodium, and calcium). The composition and structure of RHA are shown in Table S2 and Figure S1. In fact, extensive research has been carried out on the composition and structure of RHA and comparison with many conventional rubber fillers such as silica or carbon black during the last three decades. Many scientific investigations and patents have been published on this subject [1-3].

Table S1. The detailed characteristics of nature rubber (NR) and Butadiene rubber (BR).

	**NR (RSS1)**	**BR (BR9000)**
Molecular structure	$-\!\!(CH_2-\overset{CH_3}{\overset{\mid}{C}}=CH-CH_2)_{\overline{n}}$	$-\!\!(CH_2-CH=CH-CH_2)_{\overline{n}}$
Dirt content	0.05	-
Volatile matter	0.95	0.5
Ash content	0.34	0.2
Nitrogen content	0.44	-
Viscosity ML (1+4) 100℃	79.9	50
Initial plasticity	44	-
Plasticity retention index	83.1	-

Table S2. Chemical composition and physical properties of RHAs [1].

**RHA sample**	**1**	**2**	**3**	**4**	**5**	**6**	**7**
SiO_2 (%)	92.40	94.60	87.86	91.71	86.98	87.3	87.2
Al_2O_3 (%)	0.30	0.3	0.68	0.36	0.84	0.15	0.15
Fe_2O_3 (%)	0.40	0.3	0.93	0.90	0.73	0.16	0.16
CaO (%)	0.70	0.40	1.30	0.86	1.40	0.55	0.55
MgO (%)	0.30	0.30	0.35	0.31	0.57	0.35	0.35
Na_2O (%)	0.07	0.20	0.12	0.12	2.46	1.12	1.12
K2O (%)	2.54	1.30	2.37	1.67	-	3.68	3.68
Loss on ignition	2.31	1.80	-	3.13	5.14	8.55	8.55
Specific gravity (g/cm^3)	2.10	2.05	-	-	2.10	2.06	2.06
Fineness: passing 45 um (%)	-	98.20	-	-	-	99.00	99.00
Mean particle size (um)	7.40	7.15	-	0.15	7.40	-	-

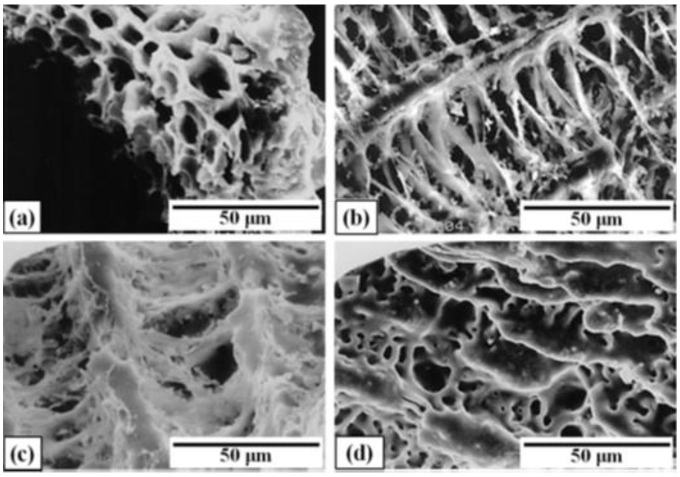

Figure S1. SEM micrographs of rice husk ashes (RHAs); (a) RHA300, (b) RHA500, (c) RHA700 and (d) RHA900 [3].

In general, the higher the pyrolysis temperature is, the lower the content of carbon and the higher the content of silica will be achieved. While carbon cannot be completely eliminated because it is trapped within the amorphous silica structure or completely coated by silica. Chen [4] reported the carbon and silica content of RHA samples which were prepared under different temperature. In air atmosphere, the silica content of RHA increases from 29.7% at 300 °C to 94.1% at 600 °C and 96.3% at 750 °C. On the contrast, the carbon content of RHA decreases from 48.8% at 300 °C to 5.4% at 600 °C and 3.2% at 750 °C, due to the oxidation of carbon to gaseous carbon dioxide. In our previous work, the final carbon and silica content of silica carbon black (SiCB) was reported which was approximately 57:43. The TG curve of SiCB in the air is shown in Figure S2.

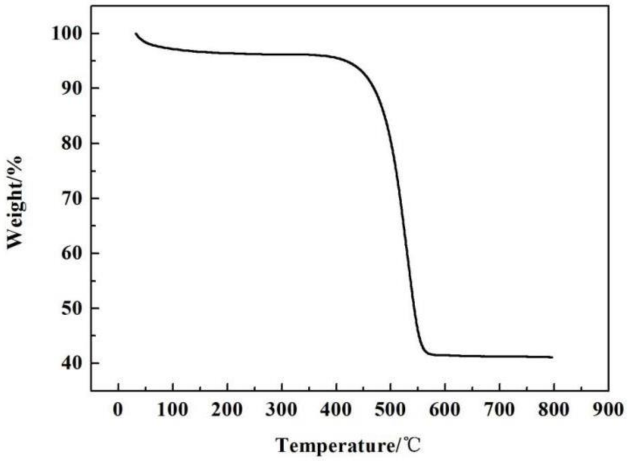

Figure S2. TG curve of SiCB in air [5].

In order to understand the state of fillers, the physical properties and the morphology of fillers are shown in Table S3 and Figure S3. The particle size of the fillers was measured by using Mastersizer-S. The samples were dispersed by ultrasound for 30 min using distilled water as the dispersant. The specific surface area of the fillers was determined by the automatic physical adsorption apparatus (ASAP 2420, Micrometrics, Atlanta, Georgia, America). The fillers were kept in an oven at 150 °C for 24 h before the adsorption isotherms test.

Table S3. Physical properties of fillers.

Fillers	Average particle size (um)	S_{BET} (m^2/g)
SiCB	1.22	239.42
S/P-100	1.06	182.16
N774	0.52	28.56

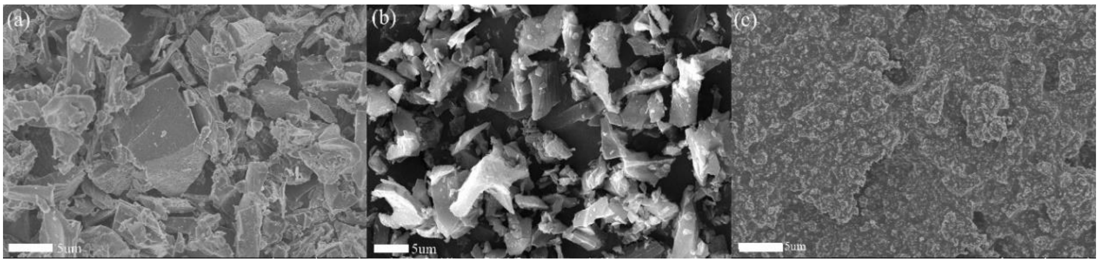

Figure S3. SEM images of (a) SiCB, (b) S/P-100 and (c) N774.

A four-factor three-level orthogonal experiment was used to optimize the PF-treated SiCB preparation conditions. Accordingly, four factors including the total amount of packing in the mixing chamber (involving the initial mixing load), mixing temperature, mixing time, and PF as the additive (based on the weight percentage of PF to SiCB).

The three different levels of four variables were chosen within a rational range for the following reasons: (1) The packing amounts are relevant to the initial mixing load. A low packing amount can result in poor interaction between PF and SiCB. Meanwhile, the torque of the machine will exceed the specified value at the high packing. (2) The temperatures have an effect on the molten state of PF. When the treated temperature lower than the softening point of the phenolic resin, the phenolic resin is not completely fused, which resulted in the agglomeration of the SiCB particles. But at a higher temperature, the flow rate of the phenolic resin is increased, a part will be peeled off from the surface of the SiCB by the action of shearing force. (3) The mixing time has an impact on the coating of PF. An appropriate time of mixing would be favorable for coating fastness. (4) The concentrations of PF have an effect on the contact between PF and SiCB, as well as the dispersion of the PF. For example, a concentration of lower than 3% would not provide enough contacts for dense coating. However, a concentration higher than 8% resulted in the agglomeration and separate phases.

The stress at 300% strain was considered as a function of these factors in the S/P-X preparation process. As shown in Table S4, the optimum treatment condition for the stress at 300% strain of composites is packing amount is 180 g, mixing temperature is 100 °C, the mixing time is 10 min, PF concentration is 3%.

Table S4. Stress at 300% strain at different treatment conditions.

Factors	Packing amount (g)	Temperature (°C)	Time (min)	PF concentration (wt %)	Stress at 300% strain (MPa)
1	160	90	5	3	2.78
2	160	95	10	5	2.85
3	160	100	15	8	2.84
4	170	90	10	8	2.64
5	170	95	15	3	2.82
6	170	100	5	5	2.80
7	180	90	15	5	2.62
8	180	95	5	8	2.80
9	180	100	10	3	3.13
m_1	2.82	2.68	2.79	**2.91**	
m_2	2.75	2.82	**2.87**	2.76	

m_3	**2.85**	**2.92**	2.76	2.76
R	0.097	0.24	0.11	0.15

m_i (i=1,2,3) stands for the mean value at the same level, e.g., m_1=(2.78 + 2.85 + 2.84)/3. R is the range of the orthogonal experiment, namely the biggest difference among each factor with the different levels of the experimental results, e.g., R=max(2.82, 2.75, 2.85)-min(2.82, 2.75, 2.85)=0.097 at factor packing amount; the two adjacent columns of m_i and R represent stress at 300% strain. The optimum level and factor are highlighted as bold [6].

In the orthogonal experiment, the larger the R is, the greater the influence of this factor will be on the experimental results. The influence of temperature on mechanical properties is larger through range analysis by comparison. Therefore, the optimum temperature was further confirmed by single-factor experiments. The preparation conditions of the fillers are shown in Table S5.

Table S5. Filler preparation conditions.

**Factors**	**Filler content (g)**	**Temperature (°C)**	**Time (min)**	**PF concentration (wt %)**
10	180	80	10	3%
11	180	90	10	3%
9	180	100	10	3%
12	180	110	10	3%
13	180	120	10	3%

To verify the effect of PF on the mechanical performance of the obtained composites, PF was added directly in the process of mixing to prepare SiCB/PF-NR/BR composites. The mechanical performance was showed in Figure S4.

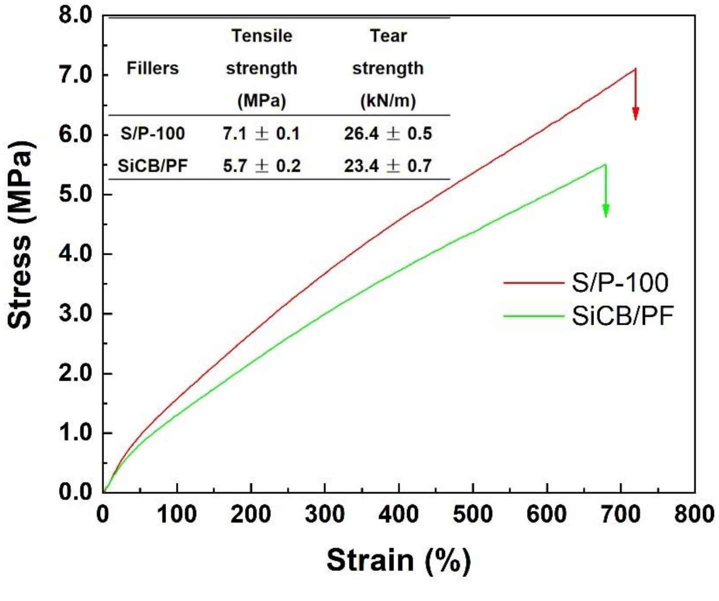

Figure S4. Uniaxial tensile curves of SiCB/PF and S/P-100 filled NR/BR composites.

References

[1] Liu, X.Y.; Chen, X.D.; Yang, L.; Chen, H.Z.; Tian, Y.M.; Wang, Z.C. A review on recent advances in the comprehensive application of rice husk ash. *Res Chem Intermed.* **2016**, *42*, 893-913.

[2] Sae-oui, P.; Rakdee, C.; Thanmathorn, P. Use of rice husk ash as filler in natural rubber vulcanizates: in comparison with other commercial fillers. *J. Appl. Polym. Sci.* **2002**, *83*, 2485-2493.

[3] Soltani, N.; Bahrami, A.; Pech-Canul, M.I. Review on the physicochemical treatments of rice husk for production of advanced materials. *Chem Eng J*. **2015**, 264, 899-935.

[4] Chen, X.G.; Lv, S.S.; Zhang, P.P.; Zhang, L.; Ye, Y. Thermal destruction of rice hull in air and nitrogen. *J. Therm. Anal. Calorim.* **2011**, *104*, 1055-1062.

[5] Zhou, Y. A study about the preparation and performance of silica carbon black/polymer composite, Jilin University, Changchun, **2017.**

[6] Zhao, J.; Wang, J.F.; Fan, L.P.; Pakdel, E.; Huang, S.; Wang, X.G. Immobilization of titanium dioxide on PAN fiber as a recyclable photocatalyst via co-dispersion solvent dip coating. *Textile Research Journal*. **2017**, 87, 570-581.
